# Triglyceride-glucose index and periodontitis: evidence from two population-based surveys

**DOI:** 10.3389/fendo.2025.1558692

**Published:** 2025-05-19

**Authors:** Jing Huang, Dan Zhang, Hua Li, Yiyun Zhang, Tianxue Long, Xiaojing Guo, Hangyu Cui, Zixuan Wei, Jun Zhao, Mingzi Li, Pangbo Wang

**Affiliations:** 1 Hand and Foot Microsurgery, Hospital of Chinese People’s Liberation Army (PLA) Land Force, Yining, China; 2 School of Nursing, Peking University, Beijing, China; 3 School of Nursing, Shanxi Medical University, Taiyuan, China; 4 Trauma Neurosurgery, Hospital of Chinese People’s Liberation Army Chinese People’s Liberation Army (PLA) Land Force, Yining, China; 5 Department of Neurosurgery and State Key Laboratory of Trauma, Burn and Combined Injury, Southwest Hospital; Chongqing Key Laboratory of Precision Neuromedicine and Neuroregeneration, Third Military Medical University (Army Medical University), Chongqing, China

**Keywords:** triglyceride-glucose index, periodontitis, NHANES, KNHANES, insulin resistance

## Abstract

**Background:**

The relationship between the Triglyceride-Glucose (TyG) index and periodontitis remains unclear. This study aims to elucidate this relationship using data from two large population-based surveys.

**Methods:**

Datasets from NHANES (2009-2014) and KNHANES (2007-2018, except for 2011) were utilized. We applied multivariate logistic regression, stratified analysis, restricted cubic splines (RCS), and subgroup analyses to examine the correlation between the TyG index and periodontitis risk. The predictive value of the TyG index was assessed using receiver operating characteristic (ROC) curves. Mediation analyses investigated variables mediating this relationship.

**Results:**

The NHANES and KNHANES cohorts included 2,511 and 16,239 participants with periodontitis, respectively. After adjusting for covariates, the TyG index was significantly associated with periodontitis risk (NHANES: OR 1.19, 95%CI: 1.07-1.34; Q2 vs. Q1, OR 1.20, 95% CI: 1.02-1.42; Q4 vs. Q1, OR 1.23, 95%CI: 1.02-1.49. KNHANES: OR 1.09, 95% CI: 1.05-1.13; Q4 vs. Q1, OR 1.09, 95%CI: 1.02-1.17, *P* for trend = 0.025). RCS analyses revealed a nonlinear relationship. ROC curves indicated that the predictive values of the TyG index were 8.24 (NHANES) and 8.69 (KNHANES). Mediation analysis showed that inflammatory (alkaline phosphatase and white blood cell) and metabolic factors (vitamin D and high-density lipoprotein cholesterol) partially mediated this association.

**Conclusions:**

The observational analysis reveals a significant association between the TyG index and the risk of periodontitis. Further studies are needed to clarify the underlying mechanisms.

## 1 Introduction

Periodontitis is a chronic disease related to microbial disorders that progressively destroys the supporting structures of the teeth, and its main clinical manifestations include clinical attachment loss, alveolar bone loss, the appearance of periodontal pockets, and gingival bleeding (1). The disease has a high prevalence, affecting nearly 62% of the population, with 23.6% classified as severe cases (2, 3). Periodontitis can lead to tooth mobility, loss, or damage, adversely affecting chewing function and overall quality of life (1). Due to a lack of obvious early symptoms, periodontitis is often overlooked until reaching irreversible stages such as tooth mobility and loss (4). Thus, identifying new biomarkers for susceptibility is crucial.

Emerging evidence indicates that the effects of periodontitis extend beyond the mouth and are linked to systemic metabolic diseases, such as metabolic syndrome, obesity, diabetes, and other chronic conditions (5, 6). Insulin resistance refers to the insensitivity of insulin target tissues to the physiological concentrations of insulin, playing a crucial role in the pathogenesis of the aforementioned metabolic diseases (7). Moreover, studies have reported that insulin resistance can exacerbate periodontal inflammation and alveolar bone loss by regulating endothelial cell function (8). Therefore, insulin resistance may serve as a potential early biomarker for periodontitis. Further investigation into the association is vital for early prevention and treatment of periodontitis.

The hyperinsulinemic-euglycemic clamp test is widely recognized as the definitive method for evaluating the body’s insulin response to glucose. However, due to its expense and complexity, it is not suitable for large-scale population studies (9). Several surrogate markers for insulin resistance have been established, among which the triglyceride-glucose (TyG) index stands out as a readily accessible and clinically promising alternative that does not require insulin measurement (10). The association between the TyG index and periodontitis has been examined in some observational studies. For example, Li et al. (11), Lee et al. (12), and Kiryowa et al. (13) reported that an elevated TyG index significantly correlated with a greater risk of periodontitis.

However, existing observational studies have several limitations such as small sample sizes, reliance on a single data source, insufficient adjustment, and lack of replication (11–13).

Thus, this study aims to explore the relationship between the TyG index and periodontitis risk using data from two large population-based surveys.

## 2 Methods

### 2.1 Study design overview

**Figure 1:**
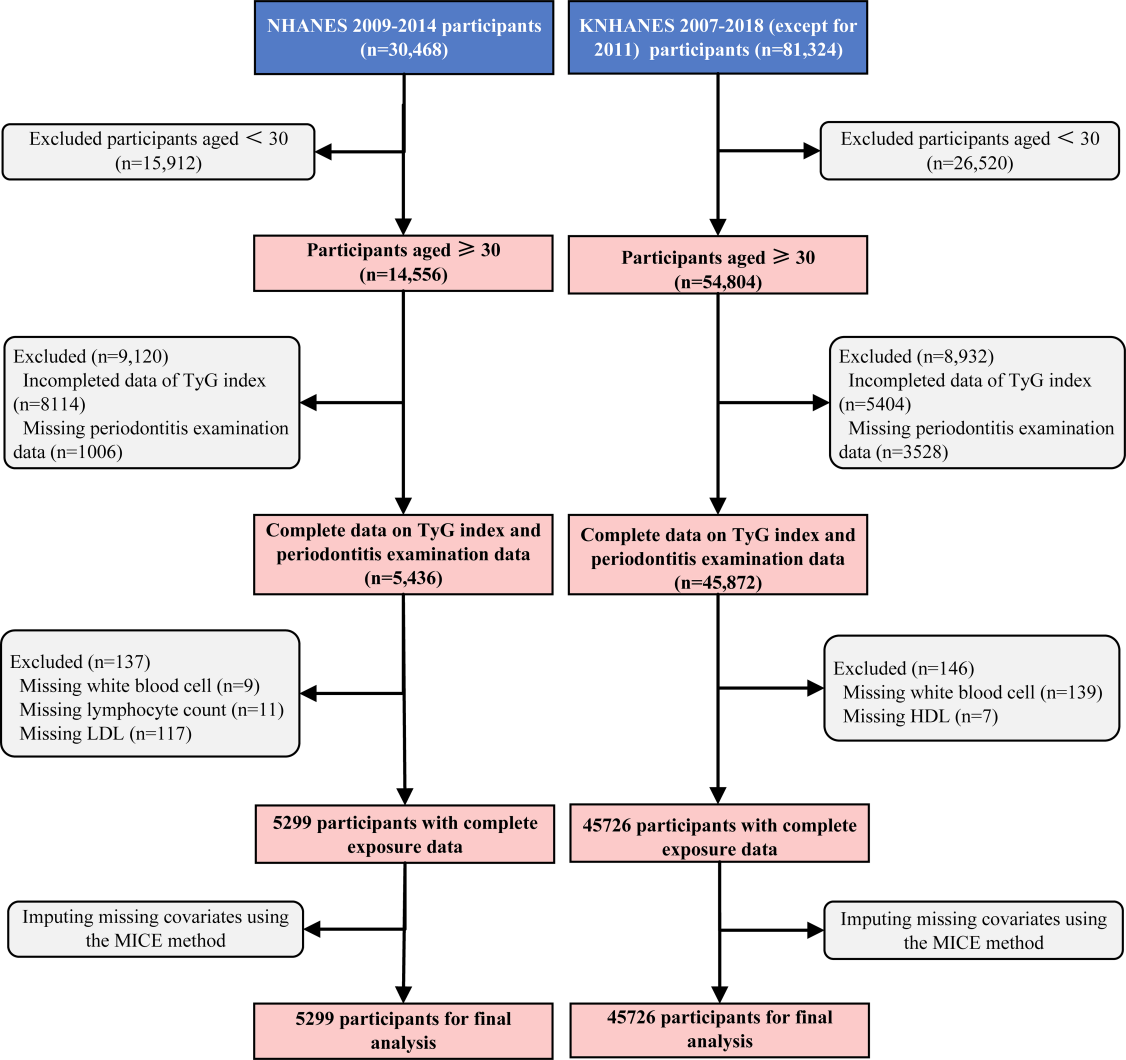
Flowchart of participants selection. **(A)**. NHANES cohort (2009-2014); **(B)**. KNHANES cohort (2007-2018, except for 2011). NHANES, National Health and Nutrition Examination Survey; KNHANES, Korea National Health and Nutrition Examination Survey; TyG, triglyceride-glucose; LDL-C, low-density lipoprotein cholesterol; HDL-C, high-density lipoprotein cholesterol; MICE, multiple imputation by chained equations.

This study consisted of two phases, with the overall design shown in **Figure 1**. We conducted a multivariable regression analysis to examine the relationship between the TyG index and periodontitis risk while accounting for various confounding factors. The data were obtained from the National Health and Nutrition Examination Survey (NHANES) and the Korean National Health and Nutrition Examination Survey (KNHANES).

### 2.2 Data and study population

#### 2.2.1 Study population 1: The NHANES

The NHANES is conducted by the National Center for Health Statistics (NCHS) to assess the health and nutritional status of adults and children in the United States (14). The survey was approved by the NCHS Ethics Review Board and participants signed an informed consent form. A total of 30,468 participants from NHANES 2009–2014 were included in this study. Potential covariates were identified based on previous study (15), including demographic characteristics (age, sex, race (Mexican American, Other Hispanic, Non-Hispanic White, Non-Hispanic Black and Other Race), educational level, marital status, and family income), lifestyle factors (body mass index (BMI), alcohol drinking, smoking status, hypertension, diabetes, and stroke), and clinical indicators (white blood cell (WBC), platelet, lymphocyte, total bilirubin, serum vitamin D, alanine aminotransferase (ALT), and high-density lipoprotein cholesterol (HDL-C)). The educational level was divided into three levels: less than 9th grade, high school graduate, and college graduate/above. The marital status was classified as married/living with a partner, widowed/separated/divorced, and others. The family income was categorized as low, middle low, middle high, and high. The exclusion criteria were as follows: (1) age < 30 years old (n = 15,912); (2) those who had incomplete data on fasting blood glucose or triglycerides (n = 8,114); (3) those who had missing data on periodontitis examination data (n = 1006); (4) those who had missing data on white blood cell (n = 9), lymphocyte count (n = 11), and low-density lipoprotein cholesterol (LDL-C) (n = 117). The variables with less than 20% were implemented through multiple imputations by chained equations (MICE). Ultimately, the final analysis comprised 5,299 participants.

#### 2.2.2 Study population 2: The KNHANES

The KNHANES is conducted by the Korea Centers for Disease Control and Prevention (KCDC) since 1998 (16). The survey was approved by the KCDC Research Ethics Review Committee, and all participants provided written informed consent. The data from KNHANES 2007-2018 (except for 2011) were utilized in this study. The following possible confounders were enrolled as covariates: demographic characteristics (age, gender, education, family income, and marital status), lifestyle factors (BMI, smoking status, alcohol drinking, hypertension, diabetes, and stroke), and clinical indicators (WBC, ALT, and HDL-C). Similarly, the classifications of educational level, marital status, and family income align with those used in the NHANES cohort. The exclusion criteria were as follows: (1) age < 30 years old (n = 26,520); (2) those who had incomplete data on fasting blood glucose or triglycerides (n = 5,404); (3) those who had missing data on periodontitis examination data (n = 3,528); (4) those who had missing data on white blood cell (n = 139), and HDL (n = 7). The missing covariates with less than 20% were implemented through MICE. Finally, the analysis comprised 45,726 participants in total.

### 2.3 Definition of TyG index

According to previous studies, the TyG index was calculated using the formula Ln [triglycerides (mg/dL) × glucose (mg/dL)/2] (17–19).

### 2.4 Ascertainment of periodontitis

In the NHANES cohort, periodontitis diagnosis relied on clinical parameters, including periodontal pocket probing depth (PD) and clinical attachment loss (AL) (20–22). Specifically, periodontal measurements were performed by licensed dental examiners at six sites per tooth (distal-facial, mid-facial, mesio-facial, distal-lingual, mid-lingual, and mesio-lingual). The following diagnostic criteria were used (see **Supplementary Table 1** for details):

No periodontitis: No evidence of mild, moderate, or severe periodontitis;

Mild periodontitis: ≥2 interproximal sites with AL ≥ 3 mm, and ≥2 interproximal sites with PD ≥ 4 mm (not on the same tooth) or one site with PD ≥ 5 mm;

Moderate periodontitis: ≥2 interproximal sites with AL ≥ 4 mm (not on the same tooth), or ≥2 interproximal sites with PD ≥ 5 mm (not on the same tooth);

Severe periodontitis: ≥2 interproximal sites with AL ≥ 6 mm (not on the same tooth) and ≥1 interproximal site with PD ≥ 5 mm.

In this study, moderate and severe periodontitis were combined into the periodontitis group, while no and mild periodontitis were classified as the reference group.

In the KNHANES cohort, periodontal status was assessed using the Community Periodontal Index (CPI), which divides the dentition into six sextants based on the FDI system (18-14, 13-23, 24-28, 38-34, 33-43, and 44-48) (23, 24). The CPI scores range from 0 to 4, where the highest score in each sextant is used to determine the periodontal health status. The criteria were as follows (see **Supplementary Table 1** for details):

No periodontitis: CPI ≤ 2 in all sextants;

Periodontitis: CPI ≥ 3 in at least one sextant.

### 2.5 Statistical analysis

Due to the intricate, multistage, and multi-stratified sampling nature of the two cohorts, sample weights were taken into account in the statistical analysis. Continuous variables were presented as mean (standard deviation, SD), while categorical variables were displayed as numbers (%). The variance test and the Kruskal-Wallis test were employed to analyze differences among multiple groups of continuous variables. For categorical variables, the Chi-square test was applied. Multivariate logistic regression was conducted to examine the relationship between the TyG index and periodontitis risk across three models. Findings were reported as odds ratios (OR) with 95% confidence intervals (CI). Model 1 did not include any covariates. Mode 2 adjusted for sociodemographic covariates including age, gender, race, education, family income, marital status, and BMI. Model 3 further adjusted for lifestyle factors and clinical indicators including alcohol drinking, smoking status, hypertension, diabetes, stroke, WBC, platelet, lymphocyte, total bilirubin, serum vitamin D, ALT, and HDL-C on the basis of Model 2.

Moreover, restricted cubic splines (RCS) were applied, adjusting for the same covariates as in model 3, to investigate the dose-response relationship between the TyG index and periodontitis risk. The participants were stratified by gender (male/female), age (30-50/>50), BMI (normal weight/overweight/obesity), hypertension (yes/no), diabetes (yes/no), and smoking status (yes/no) to investigate the association between the TyG index and periodontitis in subgroup analysis.

Further, mediation analyses were employed to examine whether the correlation between the TyG index and periodontitis could be attributed to inflammatory factors, metabolic factors, and oxidative stress biomarkers after adjusting for confounders in model 3. Based on the previous studies, we selected systemic immune-inflammation Index (SII), WBC, and alkaline phosphatase (ALP) as markers of chronic inflammation (25). Vitamin D, HDL-C, and LDL-C were employed as markers to assess metabolic changes (26). The calculation formula for the SII is as follows: SII = platelet count × neutrophil count/lymphocyte count.

The predictive value of the TyG index in both cohorts was assessed using the receiver operating characteristic (ROC) curve and the area under the curve (AUC). All statistical analyses were performed using R software (version 4.4.1). A two-sided *P* value < 0.05 was considered statistically significant.

## 3 Results

### 3.1 Baseline characteristics

**Table 1** presents the baseline characteristics of the study population. In the NHANES cohort, among the 5,299 study subjects, 2,511(47.4%) were periodontitis cases.

**Table 1:** Baseline characteristics of the study population according to periodontal variables.

Variables	NHANES (2009-2014)			*P*-value	KNHANES IV-VII			*P*-value
	Overall (n=5299)	Non-periodontitis (n=2788)	Periodontitis (n=2511)		Overall (n=45726)	Non-periodontitis (n=29487)	Periodontitis (n=16239)	
Age, years (mean ± SD)	53.6 ± 14.71	51.27 ± 15.17	56.19 ± 13.73	0.000	52.87 ± 13.79	50.38 ± 13.82	57.38 ± 12.55	0.000
Gender, n (%)				0.000				0.000
Male	2594 (49.0)	1143 (41.0)	1451 (57.8)		19716 (43.1)	11103 (37.7)	8613 (53.0)	
Female	2705 (51.0)	1645 (59.0)	1060 (42.2)		26010 (56.9)	18384 (62.3)	7626 (47.0)	
Race, n (%)				0.219				
Mexican American	720 (13.6)	285 (10.2)	435 (17.3)					
Other Hispanic	553 (10.4)	285 (10.2)	268 (10.7)					
Non-Hispanic White	2381 (44.9)	1431 (51.3)	950 (37.8)					
Non-Hispanic Black	1030 (19.4)	472 (16.9)	558 (22.2)					
Other Race	615 (11.6)	315 (11.3)	300 (11.9)					
Education, n (%)				0.000				0.000
Less than 9th grade	595 (11.2)	226 (8.1)	369 (14.7)		17375 (38.0)	9274 (31.5)	8101 (49.9)	
High school graduate	1934 (36.5)	894 (32.1)	1040 (41.4)		14230 (31.1)	9471 (32.1)	4759 (29.3)	
College graduate/above	2770 (52.3)	1668 (59.8)	1102 (43.9)		14121 (30.9)	10742 (36.4)	3379 (20.8)	
Marital status, n (%)				0.971				0.042
Married/living with partner	3412 (64.6)	1810 (64.9)	1611 (64.2)		36703 (80.3)	23819 (80.8)	12884 (79.3)	
Widowed/separated/divorced	1312 (24.8)	653 (23.4)	659 (26.2)		7011 (15.3)	4092 (13.9)	2919 (18.0)	
Others	566 (10.7)	325 (11.7)	241 (9.6)		2012 (4.4)	1576 (5.3)	426 (2.7)	
Family income, n (%)				0.000				0.000
Low	1331 (25.1)	581 (20.8)	750 (29.9)		8861 (19.4)	4768 (16.2)	4093 (25.2)	
Middle low	1323 (25.0)	642 (23.0)	681 (27.1)		11495 (25.1)	7103 (24.1)	4392 (27.0)	
Middle high	1325 (25.0)	727 (26.1)	598 (23.8)		12560 (27.5)	8495 (28.8)	4065 (25.0)	
High	1320 (24.9)	838 (30.1)	482 (19.2)		12810 (28.0)	9121 (30.9)	3689 (22.7)	
BMI, kg/m² (mean ± SD)	29.17 ± 6.71	29.20 ± 6.83	29.14 ± 6.57	0.965	23.95 ± 3.30	23.75 ± 3.30	24.31 ± 3.28	0.000
Smoke, n (%)				0.000				0.000
Yes	1009 (19.0)	431 (15.5)	578 (23.0)		11338 (24.8)	5985 (20.3)	5353 (33.0)	
No	4290 (81.0)	2357 (84.5)	1933 (77.0)		34388 (75.2)	23502 (79.7)	10886 (67.0)	
Alcohol drinking, n (%)				0.246				0.014
Yes	3876 (73.1)	2058 (73.8)	1818 (72.4)		23912 (52.3)	15294 (51.9)	8618 (53.1)	
No	1423 (26.9)	730 (26.2)	693 (27.6)		21814 (47.7)	14193 (48.1)	7621 (46.9)	
Hypertension, n (%)				0.001				0.000
Yes	4846 (91.5)	2517 (90.3)	2329 (92.8)		10924 (23.9)	5810 (19.7)	5114 (31.5)	
No	453 (8.5)	271 (9.7)	182 (7.2)		34802 (76.1)	23677 (80.3)	11125 (68.5)	
Diabetes, n (%)				0.000				0.000
Yes	845 (15.9)	383 (13.7)	462 (18.4)		4111 (9.0)	1995 (6.8)	2116 (13.0)	
No	4454 (84.1)	2405 (86.3)	2049 (81.6)		41615 (91.0)	27492 (93.2)	14123 (87.0)	
Stroke, n (%)				0.586				0.000
Yes	200 (3.8)	109 (3.9)	91 (3.6)		442 (1.0)	221 (0.7)	221 (1.4)	
No	5099 (96.2)	2679 (96.1)	2420 (96.4)		45284 (99.0)	29266 (99.3)	16018 (98.6)	
WBC, 1000 cells/ul (mean ± SD)	6.71 ± 2.39	6.64 ± 2.00	6.78 ± 2.76	0.119	6.14 ± 1.77	5.98 ± 1.70	6.41 ± 1.86	0.000
Alkaline Phosphatase, u/l (mean ± SD)	67.83 ± 22.16	66.29 ± 21.93	69.53 ± 22.30	0.000				
Platelet, 1000 cells/ul (mean ± SD)	233.82 ± 62.69	234.65 ± 60.84	232.90 ± 64.68	0.053				
Neutrophils, 1000 cells/ul (mean ± SD)	3.96 ± 1.95	3.93 ± 1.62	3.99 ± 2.27	0.452				
Lymphocyte, 1000 cells/ul (mean ± SD)	1.98 ± 0.67	1.96 ± 0.63	2.00 ± 0.70	0.060				
Serum vitamin D, nmol/l (mean ± SD)	66.38 ± 27.86	68.47 ± 28.24	64.06 ± 27.26	0.000				
Alanine Aminotransferase, U/L (mean ± SD)	25.29 ± 18.35	24.63 ± 17.17	26.02 ± 19.55	0.000	22.22 ± 18.25	21.59 ± 17.91	23.38 ± 18.79	0.000
Total bilirubin, umol/L (mean ± SD)	12.55 ± 4.82	12.44 ± 4.83	12.67 ± 4.81	0.059				
HDL, mg/dl (mean ± SD)	54.03 ± 15.80	54.64 ± 15.62	53.35 ± 15.97	0.000	49.34 ± 11.83	50.34 ± 11.94	47.53 ± 11.41	0.000

The continuous variables were presented as median (interquartile range, IQR), and the categorical variables were shown as numbers and percentages. NHANES, National Health and Nutrition Examination Survey; KNHANES, Korean National Health and Nutrition Examination Survey; BMI, body mass index; SD, standard deviation; WBC, white blood cell; HDL, high-density lipoprotein cholesterol.

The P-value in bold indicates a statistical significance.

Compared with the non-periodontitis group, participants with periodontitis were more prone to be older, male, smokers, to have a higher prevalence of chronic diseases (hypertension and diabetes), and to have lower levels of education and family income. In addition, in the periodontitis group, the levels of serum vitamin D and HDL-C were significantly lower, and the levels of ALP and ALT were significantly higher, compared to the non-periodontitis group. In the KNHANES cohort, among the 45,726 study subjects, 16,239(35.5%) were periodontitis cases. Participants with periodontitis were more likely to be older, male, obese, smokers, alcohol consumers, to have lower levels of education and family income, and to have a higher prevalence of chronic diseases (hypertension, diabetes, and stroke). Furthermore, in the periodontitis group, the levels of HDL-C were significantly lower, and the levels of total bilirubin and WBC were significantly higher, compared to the healthy group.

### 3.2 Association between the TyG index and periodontitis: results from two cohorts

**Table 2** shows the association between the TyG index and the risk of periodontitis in two cohorts. In the NHANES, the results revealed that the TyG index was positively correlated with the risk of periodontitis in the three models. In model 3, compared with Q1, a significant increase in the OR was found in Q2 (OR: 1.20; 95% Cl: 1.02-1.42; *P* = 0.028) and Q4 (OR: 1.23; 95% Cl:1.02-1.49; *P* = 0.033) after adjusting for covariates. When the TyG index was analyzed as a continuous variable, its positive correlation with periodontitis still existed across all three models. For every one-unit rise in the TyG index, the periodontitis risk increased by 19% in model 3 (*P* = 0.002).

**Table 2:** Association between the TyG index and the risk of periodontitis from the NHANES Cohort (2009-2014) and KNHANES Cohort (2007-2018, except for 2011).

Model		TyG index					*P* for trend
		Continuous	Q1	Q2	Q3	Q4	
NHANES Cohort^a^							
Model 1^a^	OR (95% CI)	**1.26(1.16-1.38)**	Ref	**1.29(1.11-1.51)**	**1.27(1.09-1.49)**	**1.41(1.21-1.65)**	**0.000**
	p value	**0.000**		**0.001**	**0.002**	**0.000**	
Model 2^a^	OR (95% CI)	**1.16(1.05-1.28)**	Ref	**1.20(1.02-1.41)**	1.12(0.95-1.33)	**1.21(1.02-1.44)**	0.096
	p value	**0.003**		**0.031**	0.164	**0.026**	
Model 3^a^	OR (95% CI)	**1.19(1.07-1.34)**	Ref	**1.20(1.02-1.42)**	1.16(0.97-1.38)	**1.23(1.02-1.49)**	0.107
	p value	**0.002**		**0.028**	0.100	**0.033**	
KNHANES Cohort^b^							
Model 1^b^	OR (95% CI)	**1.57(1.52-1.61)**	Ref	**1.38(1.31-1.47)**	**1.70(1.61-1.80)**	**2.15(2.03-2.27)**	**0.000**
	p value	**0.000**		**0.000**	**0.000**	**0.000**	
Model 2^b^	OR (95% CI)	**1.22(1.18-1.26)**	Ref	**1.07(1.01-1.14)**	**1.14(1.07-1.21)**	**1.33(1.25-1.42)**	**0.000**
	p value	**0.000**		**0.026**	**0.000**	**0.000**	
Model 3^b^	OR (95% CI)	**1.09(1.05-1.13)**	Ref	1.01(0.95-1.07)	1.01(0.95-1.08)	**1.09(1.02-1.17)**	**0.025**
	p value	**0.000**		0.86	0.769	**0.015**	

NHANES Cohort^a^:

Model 1^a^: adjusted for none.

Model 2^a^: adjusted for age, gender, race, education, family income, marital status, and body mass index.

Model 3^a^: further adjusted for smoking status, alcohol drinking, hypertension, diabetes, stroke, white blood cell, platelet, lymphocyte, total bilirubin, serum vitamin D, alanine aminotransferase, and high-density lipoprotein cholesterol.

TyG, triglyceride glucose index; NHANES, National Health and Nutrition Examination Survey; 95% CI, 95% confidence interval; OR, Odds ratios.

KNHANES Cohort^b^:

Model 1^b^: adjusted for none.

Model 2^b^: adjusted for age, gender, education, family income, marital status, and body mass index.

Model 3^b^: further adjusted for smoking status, alcohol drinking, hypertension, diabetes, stroke, white blood cell, alanine aminotransferase, and high-density lipoprotein cholesterol.

KNHANES, Korean National Health and Nutrition Examination Survey

The P-value in bold indicates a statistical significance.

Similarly, a significant positive correlation was also present in the KNHANES in all models. In detail, compared with the Q1, Q4 in model 3 had an increased risk of periodontitis, and the OR (95% CI) value was 1.09(1.02-1.17), and the *P* for trend was 0.025 (**Table 2**). When analyzed as a continuous variable, the TyG index showed a significant relationship with periodontitis in all three models. In model 3, the periodontitis risk rose by 9% for each unit increase in the TyG index (*P* = 0.000) (**Table 2**).

**Figure 2:**
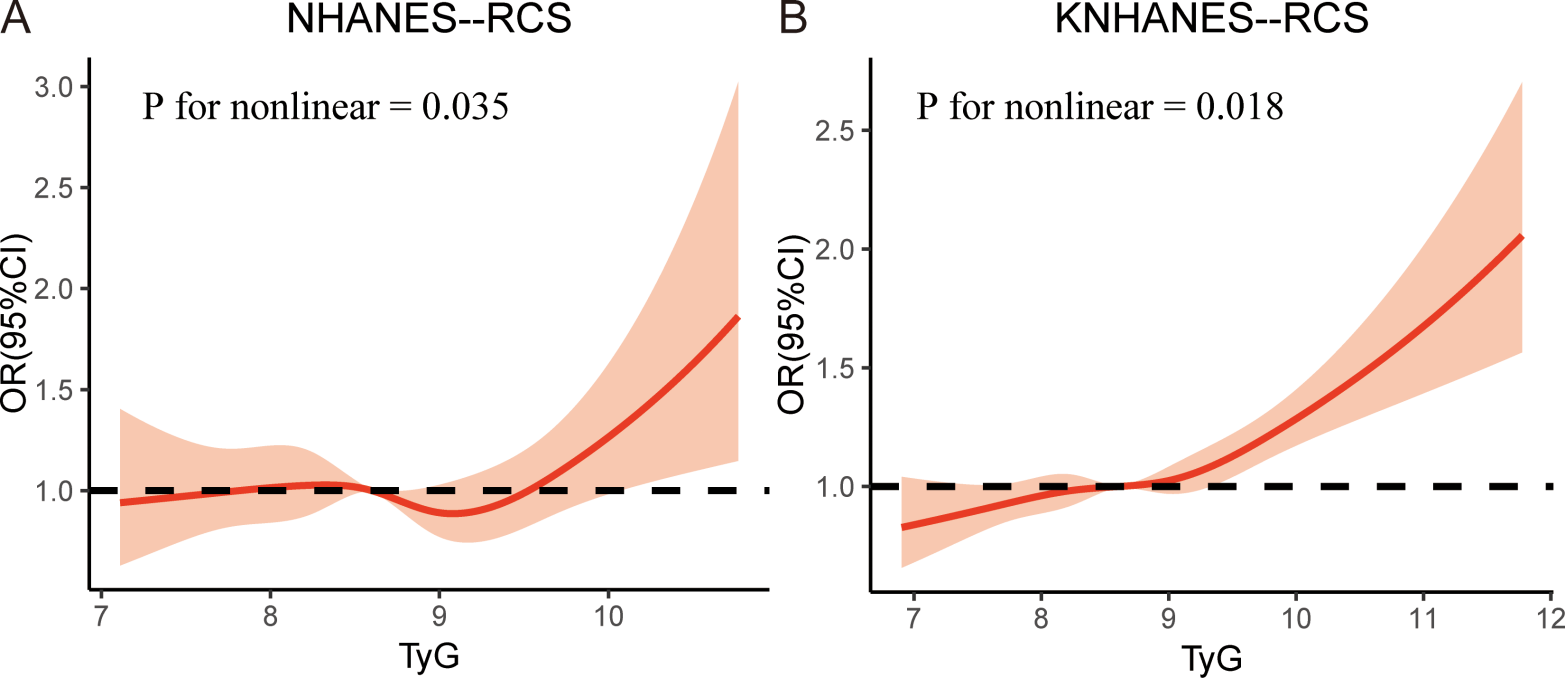
The restricted cubic spline (RCS) analysis between the TyG index and periodontitis in two cohorts. **(A)** TyG index in NHANES; **(B)** TyG index in KNHANES. NHANES, National Health and Nutrition Examination Survey; KNHANES, Korea National Health and Nutrition Examination Survey; TyG, triglyceride-glucose; OR, odds ratios.

In addition, we performed an RCS analysis to investigate the dose-response relationship between the TyG index and the risk of periodontitis. After adjusting for all covariates as in model 3, a nonlinear relationship was observed between the TyG index and the risk of periodontitis in both cohorts (*P*-nonlinear = 0.035 in NHNAES and *P*-nonlinear = 0.018 in KNHANES) (**Figure 2**).

### 3.3 Subgroup analysis

**Figure 3:**
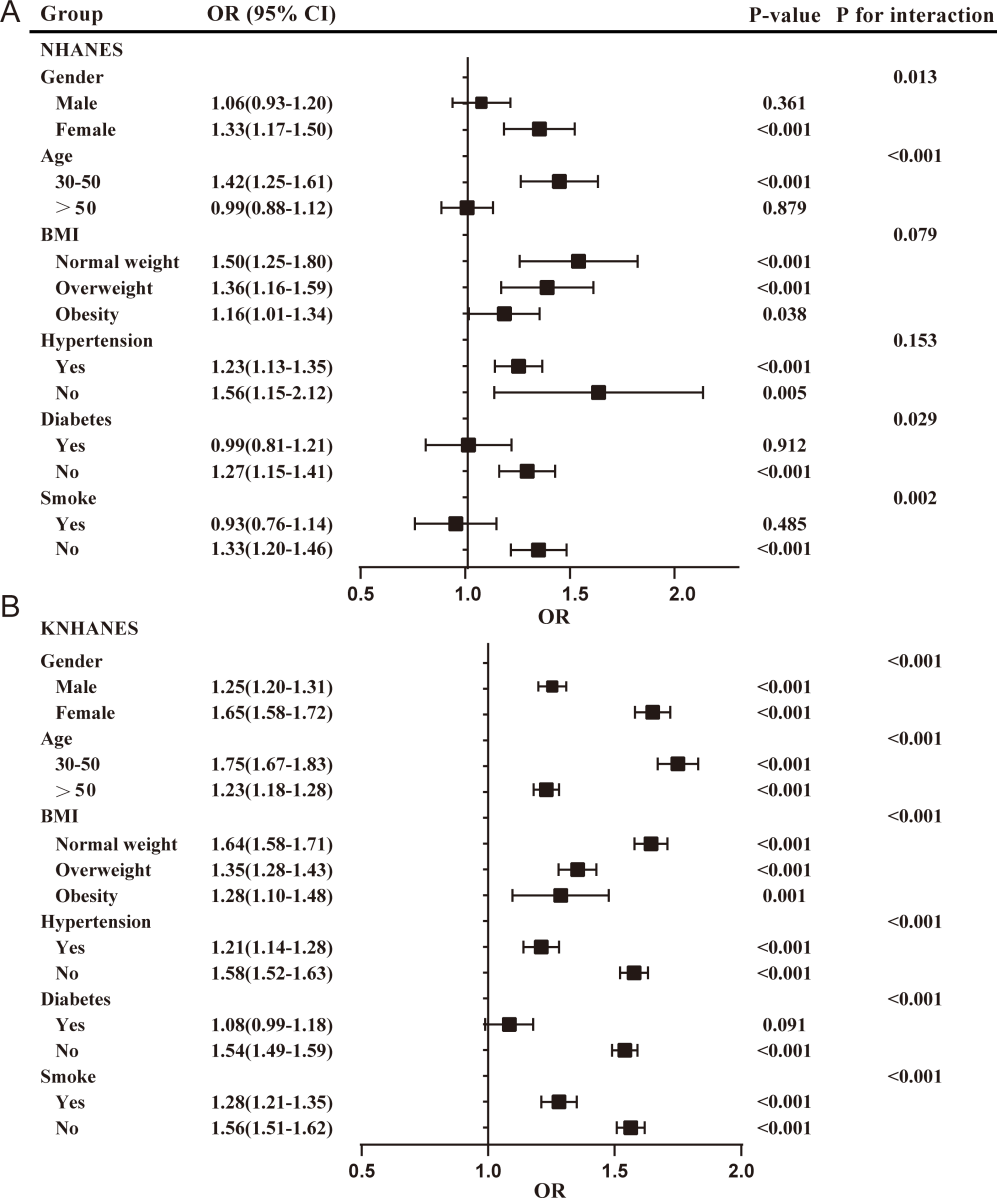
Subgroup analyses for the association between the TyG index and the risk of periodontitis in two cohorts. **(A)** TyG index in NHANES; **(B)** TyG index in KNHANES. NHANES, National Health and Nutrition Examination Survey; KNHANES, Korea National Health and Nutrition Examination Survey; BMI, body mass index; OR, odds ratios.

Subgroup analyses were conducted to estimate the associations between the TyG index and the risk of periodontitis across diverse population. The participants were stratified based on gender, age, BMI, hypertension, diabetes, and smoke. The results were presented in **Figure 3**.

In the NHANES cohort, the positive association between the TyG index and the risk of periodontitis was more pronounced in females (OR = 1.33; 95%CI: 1.17-1.50), participants younger than 50 years (OR = 1.42; 95% CI: 1.25-1.61), normal-weight (OR = 1.50; 95% CI: 1.25-1.80) and overweight participants (OR = 1.36; 95% CI: 1.16-1.59), participants without hypertension (OR=1.56; 95% CI: 1.15-2.12), participants without diabetes (OR = 1.27; 95% CI: 1.15-1.41), and non-smokers (OR = 1.33; 95% CI: 1.20-1.46). The interaction test suggested that the relationship between the TyG index and the risk of periodontitis was affected by gender (male/female), age (30-50/>50), diabetes (yes/no), and smoke (yes/no) stratification (*P* < 0.05). Females, aged 30 to 50 years, without diabetes, and who are non-smokers, may serve as effect modifiers.

In the KNHANES cohort, all subgroup analyses revealed a positive association between the TyG index and the risk of periodontitis (*P* < 0.05), except for participants with diabetes. Interaction tests indicated that the relationship between the TyG index and the risk of periodontitis was influenced by stratification through age, sex, BMI, hypertension, diabetes, and smoke. These may act as effect modifiers.

### 3.4 Mediation analysis of the TyG index and periodontitis

Inflammatory factors, metabolic factors, and oxidative stress biomarkers were identified as mediating variables to explore their effects on the relationship between the TyG index and risk of periodontitis in both cohorts.

In the NHANES cohort, the results found that inflammatory factors (ALP) and metabolic factors (vitamin D) partially mediated the association between the TyG index and the periodontitis risk (**Table 3**, **Supplementary Figure 1**). The mediation effect of ALP was 0.003 (0.001-0.01). The ALP-mediated effects of the TyG index on periodontitis was 11%. Meanwhile, the mediation effect of vitamin D was -0.002 (-0.004-0.00) and the vitamin D-mediated effects of the TyG index on periodontitis was -9.0%. Vitamin D exerted a suppressing effect on the association between the TyG index and the periodontitis risk.

**Table 3:** Mediating effects and ratios of inflammatory factors, metabolic factors, and oxidative stress biomarkers in the association between TyG index and the risk of periodontitis.

Pathways	Indirect effect	95% CI	*P*-value	Mediation proportions	95% CI	*P*-value
NHANES cohort						
Inflammatory factors						
TyG→SII→periodontitis	0.001	-0.0001-0.00	0.10	3.4%	-0.006-0.12	0.10
TyG→WBC→periodontitis	-0.0001	-0.003-0.00	0.98	-0.15%	-0.15-0.09	0.98
TyG→ALP→periodontitis	0.003	0.001-0.01	0.02	11%	0.04-0.38	0.04
Metabolic factors						
TyG→Vitamin D→periodontitis	-0.002	-0.004-0.00	0.00	-9.0%	-0.28–0.03	0.00
TyG→HDL-C→periodontitis	-0.012	-0.022-0.00	0.06	77.3%	-11.95-20.81	0.74
TyG→LDL-C→periodontitis	0.007	0.002-0.01	0.00	-40.17	-15.32-9.01	0.64
Oxidative stress factors						
TyG→ALT→periodontitis	0.003	-0.0001-0.01	0.08	-1.9%	-2.94-11.67	0.96
TyG→total bilirubin→periodontitis	-0.0003	-0.001-0.00	0.58	0.8%	-0.81-4.14	0.92
KNHANES cohort						
Inflammatory factors						
TyG→WBC→periodontitis	0.004	0.003-0.01	0.00	21.4%	0.17-0.27	0.00
Metabolic factors						
TyG→HDL-C→periodontitis	0.003	0.002-0.01	0.00	17.9%	0.10-0.26	0.00
Oxidative stress factors						
TyG→ALT→periodontitis	0.001	-0.0001-0.00	0.18	2.3%	-0.007-0.07	0.18

TyG, triglyceride glucose index; NHANES, National Health and Nutrition Examination Survey; KNHANES, Korean National Health and Nutrition Examination Survey; SII, Systemic Immune-Inflammation Index; WBC, White blood cell; ALP, Alkaline phosphatase; HDL-C, high-density lipoprotein cholesterol; LDL-C, low-density lipoprotein cholesterol; ALT, Alanine Aminotransferase.

In the KNHANES cohort, the results found that inflammatory factors (WBC) and metabolic factors (HDL-C) mediated the relationship between the TyG index and the periodontitis risk (**Table 3**, **Supplementary Figure 2**). The mediation effect of WBC was 0.004 (0.003-0.01), and the WBC-mediated effect of the TyG index on periodontitis was 21.4%. Further, the mediation effect of HDL-C was 0.003 (0.002-0.01), and the HDL-C-mediated effect of the TyG index on periodontitis was 17.9%.

### 3.5 Diagnostic efficacy of TyG index for periodontitis

**Figure 4:**
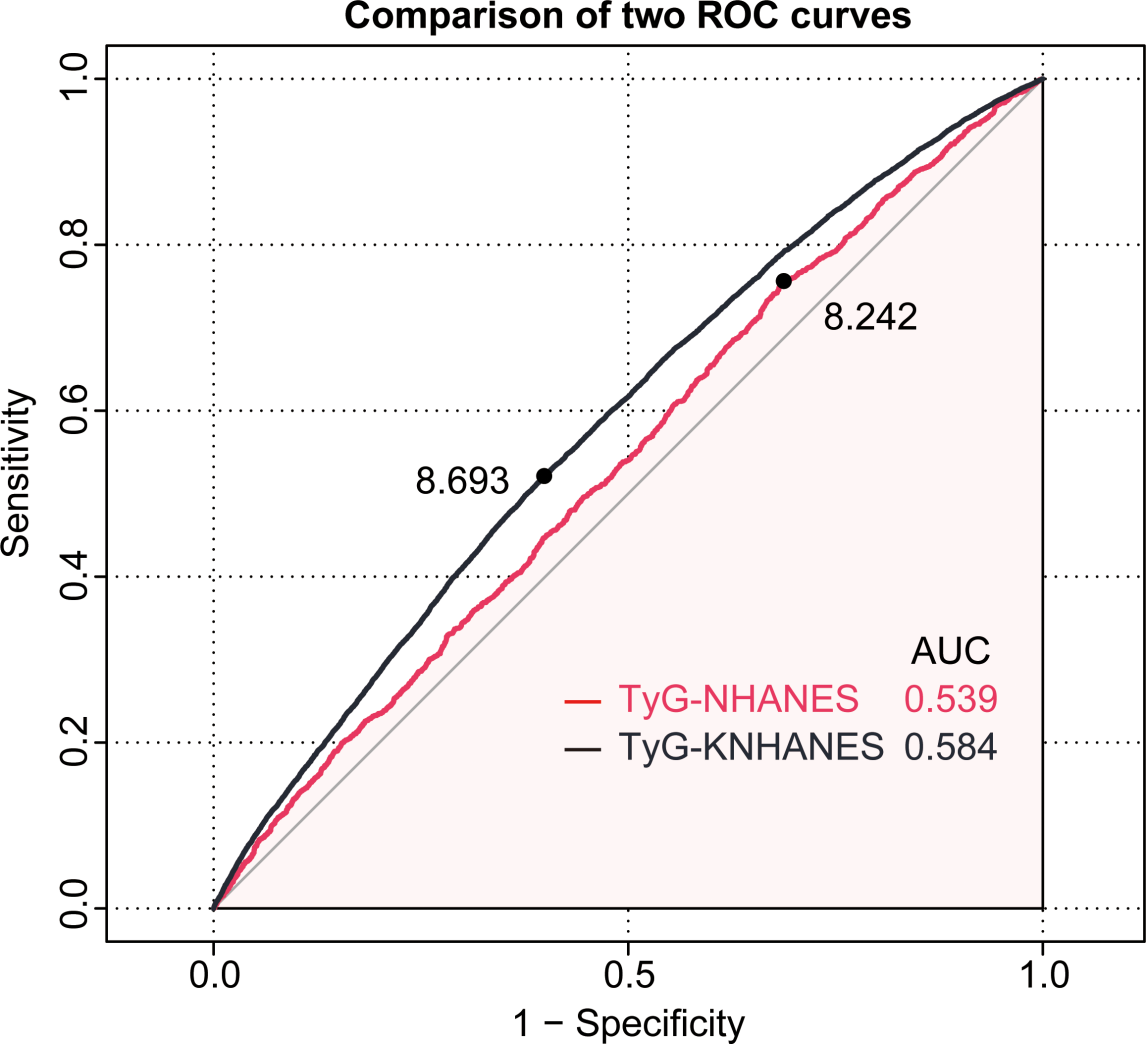
The receiver operating characteristic(ROC) curve of the TyG index for diagnosing periodontitis in two cohorts. The red line represents the NHANES cohort, while the black line represents the KNHANES cohort.

ROC curves were employed to evaluate the diagnostic effectiveness of the TyG index on patients with periodontitis. In the NHANES cohort, the cut-off value for the TyG index in the diagnosis of periodontitis was 8.24 (AUC = 0.54, 95%CI: 0.523-0.554, sensitivity = 75.6%, specificity = 31.2%) (**Figure 4**). In the KNHANES cohort, the cut-off value for the TyG index in the diagnosis of periodontitis was 8.69 (AUC = 0.58, 95% CI: 0.579-0.590, sensitivity = 52.1%, specificity = 60.1%) (**Figure 4**). AUC values above 0.5 are considered diagnostic.

## 4 Discussion

This study employed a cohort design and multi-population data to assess the association between the TyG index and periodontitis risk. We discovered that a rise in the TyG index was linked to an elevated risk of periodontitis. These associations persisted even after adjusting for covariates and exhibited a nonlinear dose-response relationship. Subgroup analysis indicated that this relationship was significant in most subgroups. Mediation analysis indicated that inflammatory factors (ALP, WBC) and metabolic factors (vitamin D, HDL-C) both play a partial mediating role in the association between the TyG index and periodontitis.

Several previous observational studies reported that the TyG index was significantly positively associated with periodontitis, aligning with the findings in this study (11–13, 27). In this study, we also found a nonlinear relationship between the TyG index and the risk of periodontitis, which is consistent with the findings reported by Li et al (11). Compared to these previous studies, our research strengthens the reliability and generalizability of the results by analyzing data from two large population databases, NHANES and KNHANES, and by using mutual validation. To the best of our knowledge, this study is the first to systematically investigate the relationship between the TyG index and periodontitis by integrating data from two large population-based surveys and employing multiple analytical approaches.

Several pathophysiological mechanisms may elucidate the potential relationship between the TyG index and periodontitis, particularly through oxidative stress and inflammation. Insulin resistance is linked to chronic inflammation, which may be critical for the development and progression of periodontitis by elevating pro-inflammatory mediators (28). It is reported that cytokines like interleukin-1 beta and tumor necrosis factor-alpha promote the recruitment of immune inflammatory cells, the production of proteases, and bone resorption. Adipokines like visfatin can promote inflammation through CC-chemokine ligand 2 and enhance matrix degradation via matrix metalloproteinase 1. These mechanisms may all intensify periodontal inflammation and tissue destruction (29–31). Moreover, insulin resistance is also associated with oxidative stress, which may exacerbate periodontitis (32, 33). Excessive accumulation of reactive oxygen species (ROS) leads to oxidative stress, which can damage tissues through DNA and protein damage, lipid peroxidation, and enzyme oxidation (34). This also can directly harm extracellular connective tissue (besides bone), resulting in attachment loss and potentially leading to periodontal tissue destruction (35). Additionally, ROS contributes to osteoclast activation, resulting in pathological bone destruction, which may cause alveolar bone resorption and damage to periodontal tissues (36). The mechanisms mentioned above may explain the significant relationship between the TyG index and periodontitis. It is important to note that insulin resistance, oxidative stress, and inflammation may all be part of metabolic syndrome, which means they could coexist rather than individually affecting periodontitis (37). Therefore, the TyG index, as a marker, may reflect a common pathological state resulting from multiple factors in its relationship with periodontitis, rather than a direct causal relationship. However, due to the complexity of metabolism, further exploration of the potential connection between the TyG index and periodontitis is needed in the future.

The findings of this study suggest that the TyG index is significantly associated with periodontitis risk in large population cohorts. Given the ease of measuring triglycerides and glucose, the TyG index could serve as a useful, low-cost tool for the early identification of individuals at higher risk of periodontitis, particularly those with metabolic disorders like insulin resistance, obesity, and diabetes. Clinicians could consider incorporating the TyG index as part of routine screenings to monitor periodontal health in patients with metabolic syndrome, potentially facilitating early intervention and preventive measures. Furthermore, our study highlights the potential role of lifestyle interventions, such as dietary modification, weight management, and physical activity, in the prevention of both metabolic disorders and periodontal disease. For individuals with elevated TyG index, focusing on improving metabolic health may not only reduce the risk of cardiovascular and diabetes complications but may also contribute to better periodontal outcomes.

In this study, we observed that inflammatory factors (such as ALP and WBC) and metabolic factors (such as HDL-C and vitamin D) partially mediate the relationship between the TyG index and periodontitis risk. Targeting these mediators could be a promising strategy to reduce the risk of periodontitis in individuals with elevated TyG index. ALP and WBC are the markers of bone metabolism and inflammation, and their elevated levels have been associated with periodontal disease progression (38, 39). Reducing systemic inflammation through lifestyle changes (e.g. increased physical activity, healthy diet rich in antioxidants, weight management, and reduction of smoking) and pharmacological interventions (e.g. statins or metformin in diabetics) could reduce inflammatory levels and, in turn, reduce the inflammatory response in periodontal tissues. HDL-C is a well-known lipid marker that has anti-inflammatory and anti-atherogenic properties. Studies suggest that low levels of HDL-C are associated with an increased risk of periodontitis (40). Improving HDL-C levels through lifestyle interventions such as increased physical activity and dietary changes (e.g., increased intake of healthy fats like omega-3 fatty acids) could help reduce the risk of periodontitis in individuals with a high TyG index. Pharmacological interventions such as niacin may also have the potential to increase HDL-C levels and offer periodontal protection. Vitamin D has a crucial role in immune regulation and bone metabolism. It is known to modulate inflammatory responses and regulate osteoclast activity, both of which are involved in periodontal bone resorption. Supplementation with vitamin D, especially in individuals with vitamin D deficiency, could be an effective intervention for reducing periodontal inflammation and supporting bone health. Ensuring adequate exposure to sunlight and dietary intake of vitamin D, as well as considering supplementation in at-risk individuals, could significantly improve periodontal outcomes.

Our subgroup analyses revealed that the association between the TyG index and periodontitis risk was stronger in females than in males. This observation warrants further exploration of potential biological and behavioral factors that may contribute to these differences. Several factors could explain why the TyG index is more strongly associated with periodontitis in females. Women are more susceptible to insulin resistance due to hormonal fluctuations, particularly the decrease in estrogen levels following menopause (41). In addition, hormonal changes during menopause are associated with increased abdominal adiposity, elevated insulin resistance, and a heightened inflammatory response (42, 43). This metabolic shift may exacerbate the inflammatory pathways involved in periodontitis, rendering women more vulnerable to its progression when experiencing elevated TyG levels.

This study utilized data from the NHANES and KNHANES databases, which offer the advantage of a large sample size. However, this study also has some limitations. First, although considerable efforts were made to adjust for various confounding factors in the observational analysis, completely eliminating the potential influence of other confounding variables remains a challenge. Second, this study lacks differentiation between aggressive and chronic periodontitis, as the NHANES and KNHANES datasets do not provide subtype-specific information for periodontitis. Since these two forms of periodontitis have different clinical features, progression rates, and underlying mechanisms, it is possible that the association between the TyG index and periodontitis risk could vary across subtypes. Aggressive periodontitis is typically characterized by rapid progression and early onset, which may make it more susceptible to metabolic disturbances such as insulin resistance and elevated TyG index. Chronic periodontitis, on the other hand, tends to progress more slowly and may involve different inflammatory and immune responses that interact with metabolic factors in distinct ways. Therefore, future research should aim to investigate the relationship between the TyG index and the different subtypes of periodontitis. This approach could also help identify more targeted preventive or therapeutic interventions for individuals at risk of different forms of periodontitis. In addition, as the NHANES and KNHANES databases primarily represent specific populations in the United States and South Korea, their demographic characteristics may restrict the generalizability of our observational findings. Finally, the cross-sectional design of this study also limited our ability to determine the causal relationship between the TyG index and periodontitis prevalence. In the future, more in-depth experimental, and prospective studies are needed to explore the causal relationship between the TyG index and periodontitis, as well as to explore its underlying mechanisms.

## 5 Conclusions

In conclusion, this study provides strong evidence of a significant association between the TyG index and the risk of periodontitis based on two large population-based surveys. The findings highlight the potential role of metabolic and inflammatory factors in this association. Further studies with more refined methodologies are needed to clarify the underlying mechanisms and to investigate whether the TyG index could serve as an early indicator for preventive strategies in periodontal health.

## Data Availability

The original contributions presented in the study are included in the article/**Supplementary Material**. Further inquiries can be directed to the corresponding author/s.

## Supplementary material

Supplementary Figure 1 Mediation effects of inflammatory factors, metabolic factors, and oxidative stress biomarkers in the associations of the TyG index and the risk of periodontitis in the NHANES cohort. SII, systemic immune-inflammation index; WBC, white blood cell; ALP, Alkaline Phosphatase; ALT, Alanine Aminotransferase; LDL-C, low-density lipoprotein cholesterol; HDL-C, high-density lipoprotein cholesterol.

Supplementary Figure 2 Mediation effects of inflammatory factors, metabolic factors, and oxidative stress biomarkers in the associations of the TyG index and the risk of periodontitis in the KNHANES cohort. WBC, white blood cell; HDL-C, high-density lipoprotein cholesterol; ALT, Alanine Aminotransferase.

Supplementary Table 1 Diagnostic criteria for periodontitis in NHANES and KNHANES NHANES, National Health and Nutrition Examination Survey; KNHANES, Korean National Health and Nutrition Examination Survey; CPI, Community Periodontal Index; PD, Pocket probing depth; AL, Attachment Loss.
