# Correction: Triglyceride-glucose index and periodontitis: evidence from two population-based surveys

**DOI:** 10.3389/fendo.2025.1686929

**Published:** 2025-09-05

**Authors:** Jing Huang, Dan Zhang, Hua Li, Yiyun Zhang, Tianxue Long, Xiaojing Guo, Hangyu Cui, Zixuan Wei, Jun Zhao, Mingzi Li, Pangbo Wang

**Affiliations:** ^1^ School of Nursing, Peking University, Beijing, China; ^2^ School of Nursing, Shanxi Medical University, Taiyuan, China; ^3^ Trauma Neurosurgery, NO. 946 Hospital of Chinese People’s Liberation Army Chinese People’s Liberation Army (PLA) Land Force, Yining, China; ^4^ Department of Neurosurgery and State Key Laboratory of Trauma, Burn and Combined Injury, Southwest Hospital; Chongqing Key Laboratory of Precision Neuromedicine and Neuroregeneration, Third Military Medical University (Army Medical University), Chongqing, China; ^5^ Hand and foot microsurgery, NO. 946 Hospital of Chinese People’s Liberation Army (PLA) Land Force, Yining, China

**Keywords:** triglyceride-glucose index, periodontitis, NHANES, KNHANES, insulin resistance

In the published article, there was a mistake in the **Funding** statement. The original **Funding** statement read: “The National Natural Science Foundation of China (72074008), the International Institute of Population Health of Peking University Health Science Center (JKCJ202304), and the Peking University Nursing Discipline Research and Development Fund (LJRC23YB03) supported this study.”

The correct **Funding** statement reads:

“The author(s) declare that no financial support was received for the research, and/or publication of this article.”

The original version of this article has been updated.

