# Correction: Triglyceride-glucose index and periodontitis: evidence from two population-based surveys

**DOI:** 10.3389/fendo.2026.1775726

**Published:** 2026-01-23

**Authors:** Jing Huang, Dan Zhang, Hua Li, Yiyun Zhang, Tianxue Long, Xiaojing Guo, Hangyu Cui, Zixuan Wei, Jun Zhao, Mingzi Li, Pangbo Wang

**Affiliations:** 1 Hand and Foot Microsurgery, Hospital of Chinese People’s Liberation Army (PLA) Land Force, Yining, China; 2 School of Nursing, Peking University, Beijing, China; 3 School of Nursing, Shanxi Medical University, Taiyuan, China; 4 Trauma Neurosurgery, Hospital of Chinese People’s Liberation Army Chinese People’s Liberation Army (PLA) Land Force, Yining, China; 5 Department of Neurosurgery and State Key Laboratory of Trauma, Burn and Combined Injury, Southwest Hospital; Chongqing Key Laboratory of Precision Neuromedicine and Neuroregeneration, Third Military Medical University (Army Medical University), Chongqing, China

**Keywords:** triglyceride-glucose index, periodontitis, NHANES, KNHANES, insulin resistance

Affiliations were erroneously assigned in the original version of the article. The correct author affiliations are now provided as follows:

Jing Huang^2^, Dan Zhang^2^, Hua Li^2^, Yiyun Zhang^2^, Tianxue Long^2^, Xiaojing Guo^2^, Hangyu Cui^2^, Zixuan Wei^3^, Jun Zhao^4^, Mingzi Li^2*^ and Pangbo Wang^1,5*^

Correct Affiliations of all authors as they should appear:

^1^Hand and Foot Microsurgery, Hospital of Chinese People’s Liberation Army (PLA) Land Force, Yining, China

^2^School of Nursing, Peking University, Beijing, China

^3^School of Nursing, Shanxi Medical University, Taiyuan, China

^4^Trauma Neurosurgery, Hospital of Chinese People’s Liberation Army Chinese People’s Liberation Army (PLA) Land Force, Yining, China

^5^Department of Neurosurgery and State Key Laboratory of Trauma, Burn and Combined Injury, Southwest Hospital; Chongqing Key Laboratory of Precision Neuromedicine and Neuroregeneration, Third Military Medical University (Army Medical University), Chongqing, China

The original version of this article has been updated to reflect the correct author-affiliation assignments. The conflict of interest statement remains unchanged.

