# Supplementary figures and images for: Triglyceride-glucose index and periodontitis: evidence from two population-based surveys
Source: Front Endocrinol (Lausanne). 2025 May 19;16:1558692. doi: 10.3389/fendo.2025.1558692 (PMC12127200; doi:10.3389/fendo.2025.1558692)

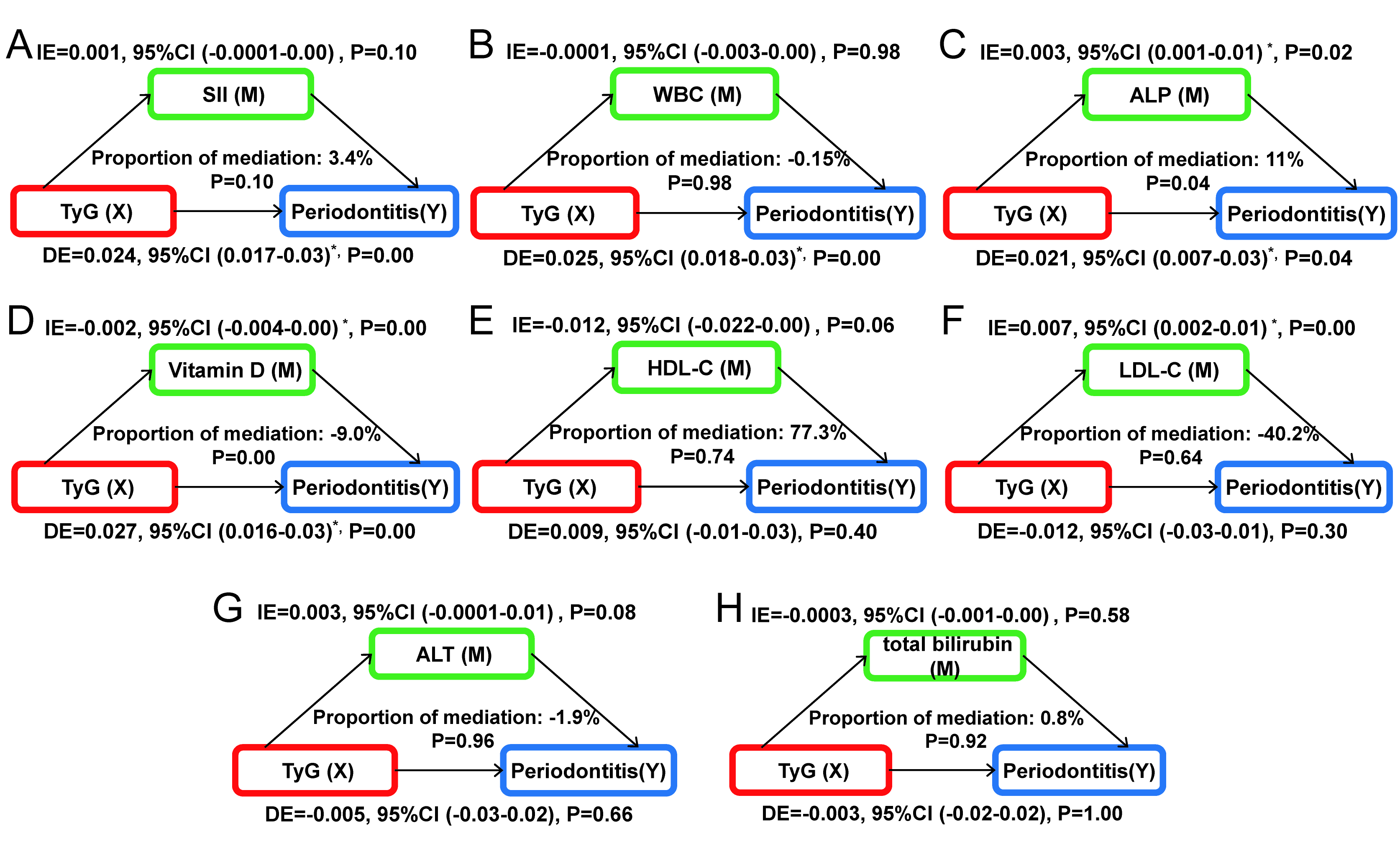

Supplement: Supplementary Figure 1 — Mediation effects of inflammatory factors, metabolic factors, and oxidative stress biomarkers in the associations of the TyG index and the risk of periodontitis in the NHANES cohort. SII, systemic immune-inflammation index; WBC, white blood cell; ALP, Alkaline Phosphatase; ALT, Alanine Aminotransferase; LDL-C, low-density lipoprotein cholesterol; HDL-C, high-density lipoprotein cholesterol. [file Image1.tif]

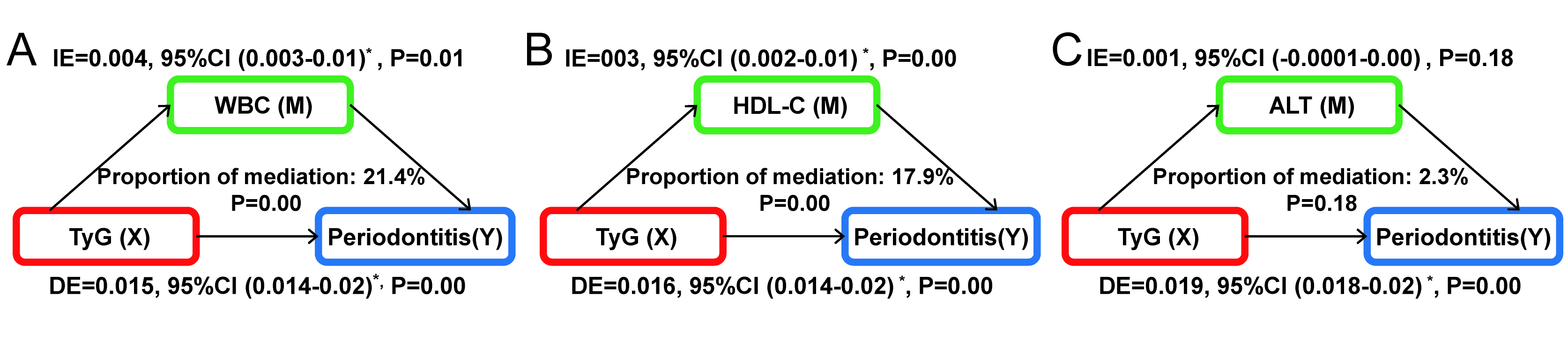

Supplement: Supplementary Figure 2 — Mediation effects of inflammatory factors, metabolic factors, and oxidative stress biomarkers in the associations of the TyG index and the risk of periodontitis in the KNHANES cohort. WBC, white blood cell; HDL-C, high-density lipoprotein cholesterol; ALT, Alanine Aminotransferase. [file Image2.tif]
